# Studies on Quality Markers of Kaihoujian Spray for Anti-Inflammation Based on Gray Correlation Analysis Strategy

**DOI:** 10.1155/2022/1273066

**Published:** 2022-04-20

**Authors:** Jinpeng Chen, Yi Liu, Xiaohong Gai, Qing Ye, Siyu Zhou, Chengwang Tian, Tiejun Zhang

**Affiliations:** ^1^Tianjin Institute of Pharmaceutical Research, Tianjin, China; ^2^Key Laboratory of TCM Quality Markers, Tianjin, China; ^3^State Key Laboratory of Drug Delivery and Pharmacokinetics, Tianjin, China

## Abstract

Kaihoujian spray (KHJ) was originated from the classical prescription of Miao medicine, which was commonly used for acute and chronic pharyngitis. The prescription was composed of Sophorae Tonkinensis Radix, Ardisiae Radix, Cicadae Periostracum, and menthol. However, in previous literature, only clinical studies have been reported. The Quality Marker (Q-Markers) of KHJ on anti-inflammation has not been clearly elucidated. In this study, a gray correlation analysis strategy combined with network pharmacology analysis was established for the investigation of Q-Markers in KHJ. A total of 52 components were identified or tentatively characterized in KHJ, including alkaloids, saponins, bergenin, flavonoids, amino acids, and their derivatives. Furthermore, regularity of recipe composition and gray correlation analysis revealed that the correlation degree of all peaks was greater than 0.5. The ranking of correlation degree was peak 1 > 6>9 > 8>7 > 10>4 > 5>11 > 3>2. Among them, peaks 2, 4, 5, 6, 8, 9, and 11 were identified as anagyrine, matrine, sophocarpine, norbergenin, bengenin, 11-O-galloylbergenin, and trifolirhizin. The network pharmacology analysis revealed that EGFR, MMP9, MMP3, MMP1, and PTGS2 were the main targets of KHJ. Bergenin, matrine, sophocarpine, calycosin, and trifolirhizin were the main anti-inflammatory active ingredient in KHJ. These results proposed that bergenin, sophocarpidine, sophocarpine, and trifolirhizin could be the Q-Markers of KHJ on anti-inflammation. The process of discovering the Q-Markers would provide a promising method of quality control on KHJ.

## 1. Introduction

As a significant portion of traditional Chinese medicine (TCM), Miao medicine has a long history of three or four thousand years. It is generally considered to be mysterious and magical and has its own system, especially famous for its external treatment of internal diseases [1]. Miao herb formulation (MHF) is a valuable medical experience accumulated by Miao folk in their long-term production activities and the practice of fighting against diseases and injuries. They have a profound understanding of etiology, elements, disease diagnosis, treatment, and prevention and have many unique features in clinical prescription and medication [2]. Their abundant medical experience has enriched the culture and become an important part of TCM.

However, similar to TCM, MHF also has many problems, such as unclear material basis and index components. Although some MHF were included in Chinese Pharmacopoeia, the quality standards were only established on the basis of their major components. Whether the index component was related to its efficacy was still dubious.

Fortunately, the concept of Quality Marker (Q-Marker) was established by Liu et al. [3] for the development and improvement of the quality of TCM. The candidates for Q-Markers should meet these criteria [3, 4]: (1) The candidates should exist in original materials, TCM products, or formed during processing and preparation. (2) The candidates should be unique to some herbs and not derived from other herbs. (3) The candidates should have definite chemical structures and biological activity. (4) The candidates could be qualitatively and quantitatively identified. (5) The candidates should follow the principle of TCM. For the past few years, numerous studies on Q-Markers have been published [5–14]. However, how to discover and verify the Q-Marker was still a serious challenge.

Kaihoujian spray (KHJ) was originated from the classical prescription of Miao medicine, which was a commonly used Chinese patent medicine for children with acute and chronic pharyngitis, and produced by Guizhou Sanli Pharmaceutical Limited by Share Ltd. The prescription was composed of Sophorae Tonkinensis Radix, Ardisiae Radix, Cicadae Periostracum, and menthol. According to the previous literature, Sophorae Tonkinensis Radix has the effects of anti-inflammation [15], antivirus [16], inhibiting bacteria [17], improving immunity [18], and so on. Ardisiae Radix has the effects of bacteriostasis [19], analgesia [20], antivirus [21], and so on. Cicadae Periostracum has the effects of antiallergic [22], antitussive and antiasthmatic [23], bacteriostatic [24], and so on. Menthol has the effects of analgesic [25], osmotic [26], and so on. Kaihoujian spray can directly act on oral mucosa and avoid first-pass effect without gastrointestinal absorption and has the clinical advantages of fast onset, high bioavailability, small side effects, short course of treatment, convenient medication, and high patient compliance [27]. At present, the Q-Markers of KHJ on anti-inflammation have not been clearly elucidated. Only clinical studies have been reported in previous literature. Hence, it was necessary to develop a strategy to discover and validate the Q-Markers of KHJ on anti-inflammation. In this study, a gray correlation analysis strategy combined with network pharmacology analysis was established for the investigation of Q-Markers on KHJ. The results showed that bergenin, sophocarpidine, sophocarpine, and trifolirhizin should be the Q-Markers of KHJ on anti-inflammation. The process of discovering the Q-Markers would provide a promising method of quality control on KHJ.

## 2. Materials and Methods

### 2.1. Materials and Chemicals

The Sophorae Tonkinensis Radix (the dried root of *Sophora tonkinensis* Gagnep.), Ardisiae Radix (the dried root of *Ardisia crenata* Sims.), Cicadae Periostracum (the dried shell of *Cryptotympana pustulata fabricius*), and menthol were provided by Guizhou Sanli Pharmaceutical Limited by Share Ltd. and identified by the Researcher Chengwang Tian. The voucher specimens (STR-2019, AR-2019, CPS-2019, and MEL-2019) were stored in herbaria at Tianjin Institute of Pharmaceutical Research, China.

(4,5-Dimethylthiazol-2-yl)-2,5-diphenyl tetrazolium bromide (MTT) and lipopolysaccharides (LPSs) were purchased from Beijing Solarbio Science & Technology Co., Ltd. The Griess reagents were purchased from Tianjin Guangfu Chemical Research Institute. Dulbecco's modified eagle's medium (DMEM) and Fetal Bovine Serum (FBS) were purchased from Gibco Ltd. All organic solvents used in this study were of HPLC grade and purchased from Concord Technology Co., Ltd. Pure distilled water was purchased from Wahaha Group Co., Ltd. (Hangzhou, China).

### 2.2. Preparation of Samples

The whole prescription sample was prepared according to the method for KHJ Spray Standard published by National Medical Products Administration in 2002. In a brief description, Sophorae Tonkinensis Radix (250 g), Ardisiae Radix (250 g), and Cicadae Periostracum (250 g) were refluxed with pure water twice (1 : 10, w/v, 2 h each). After evaporation of the solvent in vacuo, ethanol was added to the residues until the ethanol content reached 80%. While kept standing for 24 hours, the solvent was filtered and evaporated in vacuo. The crude extract was mixed with menthol (1 g). Samples for regularity of recipe composition (KHJ-1∼14) were prepared as the above method, except for the assigned herbs. The ingredients of each sample are shown in Table 1.

### 2.3. HPLC/Q-Tof-MS/MS Analysis of KHJ

The sample for HPLC/Q-TOF-MS/MS analysis was prepared as follows: approximately 0.1 g of the whole prescription sample (KHJ-15) was dissolved and diluted to 10 mL by 40% methanol. The sample was filtered through a 0.22 *μ*m membrane filter and then injected 10 *μ*L filtrate into the HPLC system for analysis. Chromatographic separation was carried out on an ultimate Plus C18 column (4.6 × 250 mm,5 *μ*m). The mobile phase was optimized as 0.1% aqueous formic acid (A) and acetonitrile (B), and the gradient of elution was as follows: 0–15 min, 3%–8% B; 15–25 min, 8%–15% B; 25–35 min, 15%–22% B; 35–45 min, 22%–24% B; 45–75 min, 24–50% B. The flow rate was 1.0 mL/min, and the column temperature was held at 30°C. The optimum absorbed wavelength was selected as 210 nm according to the favorable resolution and multiple chromatographic peaks.

The mass spectrometry analysis was obtained on a Sciex X500 R QTOF mass spectrometer equipped with an electrospray interface (ESI) source (AB Sciex, Framingham, MA, USA). Positive and negative ion modes were used for detection, the capillary voltages were 5500 V and 4500 V, and the cleavage voltage was 50 V and 80 V, respectively. The curtain gas was 35 PSI, and the atomizing temperature was 600°C. The mass data were achieved in the range of *m/z* from 50 to 1800 Da with a response value of more than 100 cps of the four highest peaks for secondary mass spectrum scanning. Data were collected and analyzed by analyst software SCIEX OS 1.4.

### 2.4. HPLC-DAD Analysis for Samples of Regularity of Recipe Composition

The analysis of the regularity of recipe composition samples (KHJ-1∼14) was performed by the Waters e2695 (United States) with a PDA detector. The gradient of elution for mobile phase, optimum absorbed wavelength, flow rate, chromatography column, and column temperature were set as HPLC/Q-TOF-MS/MS method.

### 2.5. Cell Culture and Cytotoxicity

RAW264.7 cells were purchased from Procell Life Science & Technology Co., Ltd. (Wuhan, China). Cells were cultured in DMEM, including 10% FBS and 2% penicillin-streptomycin solution at 37°C in humidified 5% CO2 atmosphere. Cells were made to suspension and diluted to 1 × 105 cells/mL by DMEM containing 10% FBS. Cytotoxicity of the samples was determined by MTT assay.

### 2.6. Inhibition of NO Production in LPS-Induced RAW264.7 Cells

RAW264.7 cells were incubated and divided into several groups. After stimulation with and without LPS (2 mg/mL) for 24 hours, the supernatant of media was collected for NO production analysis. 50 *μ*L supernatant mixed with 50 *μ*L Griess reagent was incubated in the dark for 10 min at 37°C. The OD value of each well was measured by a microplate reader at 540 nm. The concentration of NO was determined by the standard curve from sodium nitrite.

### 2.7. Statistical Analysis

The measurement data were analyzed by IBM SPSS 23.0 (USA) and expressed as means ± SD (*n* = 3). The data satisfying normality and homogeneity of variance were analyzed by one-way ANOVA, and the comparison between groups was performed by the LSD method. If the data do not meet the homogeneity of variance, using *K* independent samples nonparametric test, *P* < 0.05 has statistical significance.

### 2.8. Network Pharmacology Analysis

All the identified compounds in HPLC/Q-Tof-MS/MS analysis of KHJ were selected for the target compounds. Then these target compounds were introduced into a SwissTargetPrediction database (https://www.swisstargetprediction.ch) to predict an action target of the active compound. The TCMSP database (https://tcmspw.com/tcmsp.php) was searched for the possible targets of the active ingredients of KHJ, and then the Dragbank database (https://www.drugbank.ca/) and Uniprot database (http://www.uniprot.org) were searched for the target GeneSymbol. Taking “inflammation” as the keyword, we searched the DigSee database (http://digsee.com) to obtain the targets related to anti-inflammatory and screened the inflammatory targets with a correlation degree greater than 0.8.

The data of active compounds and inflammatory targets screened above were sorted and imported into Cytoscape 3.7.0 software to construct the active ingredient-disease target network, and the network topology was analyzed by Network Analyzer. The intersection targets of drugs and diseases were imported into the STRING database (https://www.string-db.org), the confidence value was set to 0.4, the target interaction data were exported and processed by Excel and then imported into Cytoscape 3.7.0 to realize visualization, and the topology analysis of PPI network was carried out. Through the ClueGO, gene ontology (GO) analysis was carried out on the core target of KHJ under the conditions of number of genes = 3 and min percentage = 4.0. *Homo sapiens*, overlap >3, *P* < 0.01, and enrichment >1.5 were selected as screening conditions for GO and KEGG analysis. The pathway with lower *P* value and more enriched genes was screened, the GO analysis was drawn by *R* language ggplot2 software package, and the bubble map of KEGG analysis was drawn by Origin Pro 2021 software. Pathways with a smaller *P* value and more enriched genes were screened, the data of drug flavor, components, targets, and pathways were sorted out and introduced into Cytoscape 3.7.0 software to construct the network of drug flavor-component-target-pathway, and the network topology was analyzed.

## 3. Results and Discussion

### 3.1. Analysis of Chemical Ingredients in KHJ

**Figure 1:**
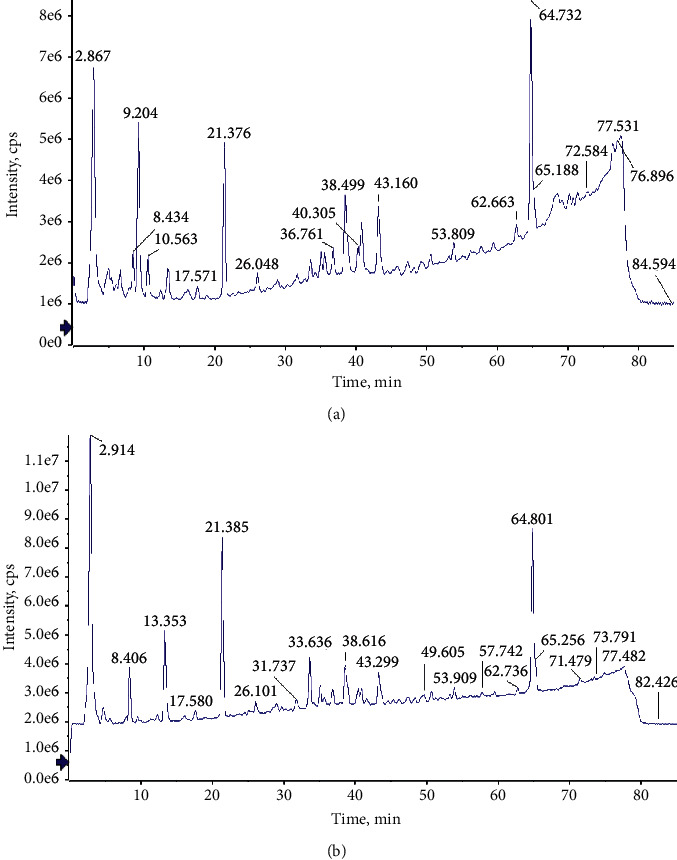
Total ion chromatogram (TIC) of KHJ at positive (a) and negative (b) ion mode.

**Figure 2:**
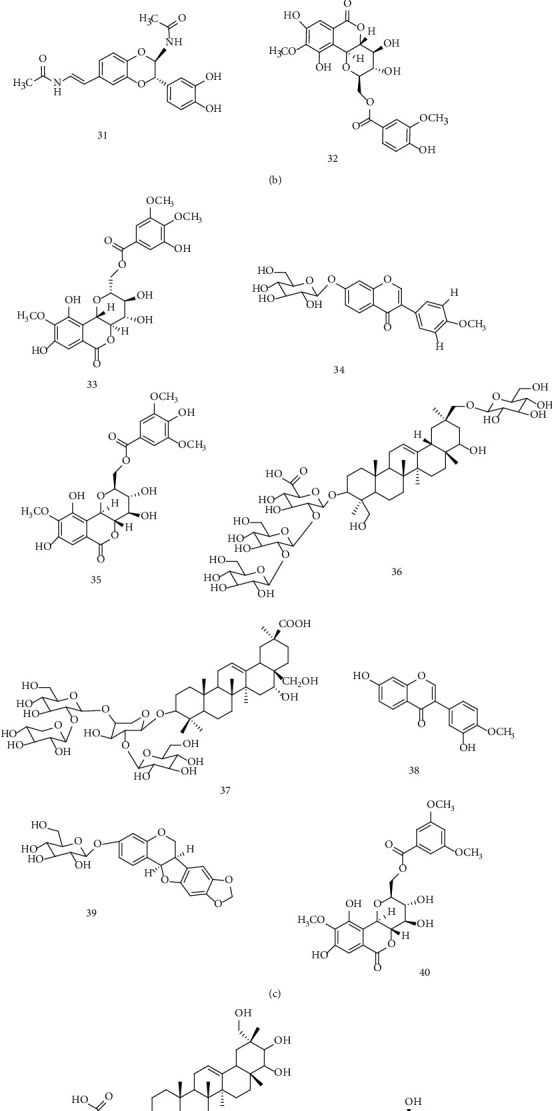
Chemical structures identified in KHJ.

The total ion chromatography (TIC) of KHJ in positive and negative modes is shown in Figure 1. A total of 52 components were identified or tentatively characterized in KHJ, including alkaloids, saponins, bergenin, flavonoids, amino acids, and their derivatives. Among them, 32 compounds (alkaloids, flavonoids, saponins, etc.) were derived from Sophorae Tonkinensis Radix, 14 compounds (coumarins, saponins, etc.) were derived from Ardisiae Radix, and 6 compounds (amino acids) were derived from Cicadae Periostracum. The identification of these compounds was mainly based on the comparison with literature, including the retention time and fragment ion. The detailed information, including chemical formula, retention time, mass value, mass error, fragment ion, and botanical source, is shown in Table 2. The exact structures are shown in Figure 2.

#### 3.1.1. Identification of Alkaloids in KHJ

**Figure 3:**
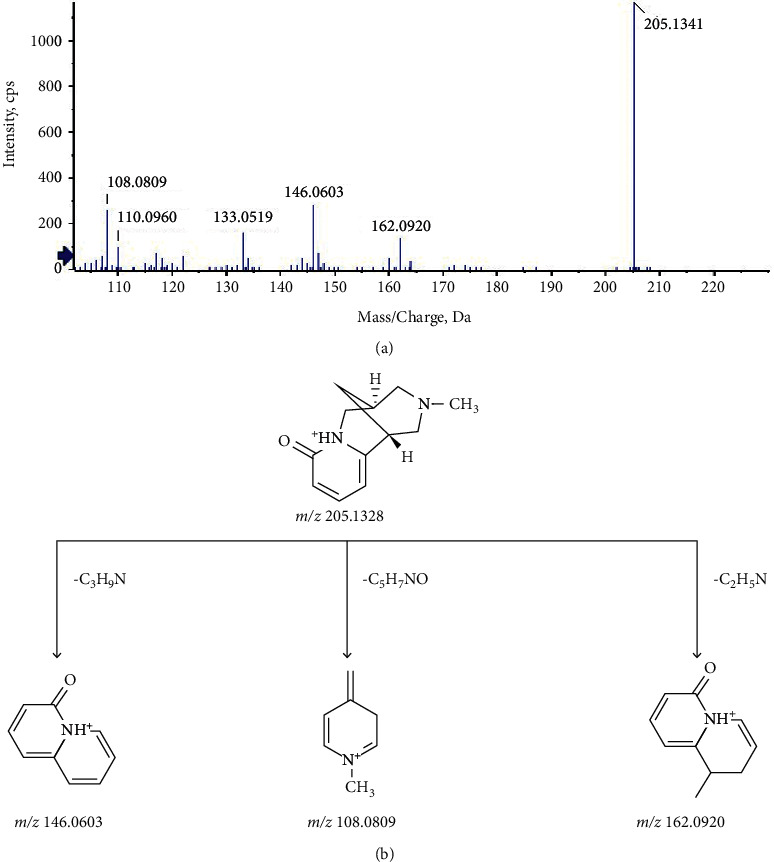
The mass spectrum and the cleavage rule of the N-methylcytisine.

Fifteen alkaloids from Sophorae Tonkinensis Radix were identified in KHJ. The main types of them were matrine and cytisine, and all of them could yield quasimolecular ions [M + H]^+^. Take N-methylcytisine (compound 1) as an example to illustrate the analytic process of alkaloids. The quasimolecular ion [M + H]^+^ at *m/*z 205.1 corresponded to the formula C_12_H_16_N_2_O. The mass spectrum fragment ion *m/z* 146.0603 [M + H–C_3_H_9_N]^+^ was the characteristic fragment ion of the cytisine alkaloid, and *m/z* 108.0809 [M + H–C_5_H_7_NO]^+^ was generated by the cleavage and rearrangement of the parent ionic bond C6–C7/C1–C10. In combination with the literature [28], compound 1 was presumed to be N-methylcytisine. The mass spectrum and the cleavage rule of the N-methylcytisine are shown in Figure 3.

#### 3.1.2. Identification of Bergenin Derivatives in KHJ

Bergenin and its derivatives are the main effective constituents of Ardisiae Radix and have the effect of relieving cough by inhibiting the cough center. In this study, seven bergenin and its derivatives from Ardisiae Radix were identified in KHJ.

**Figure 4:**
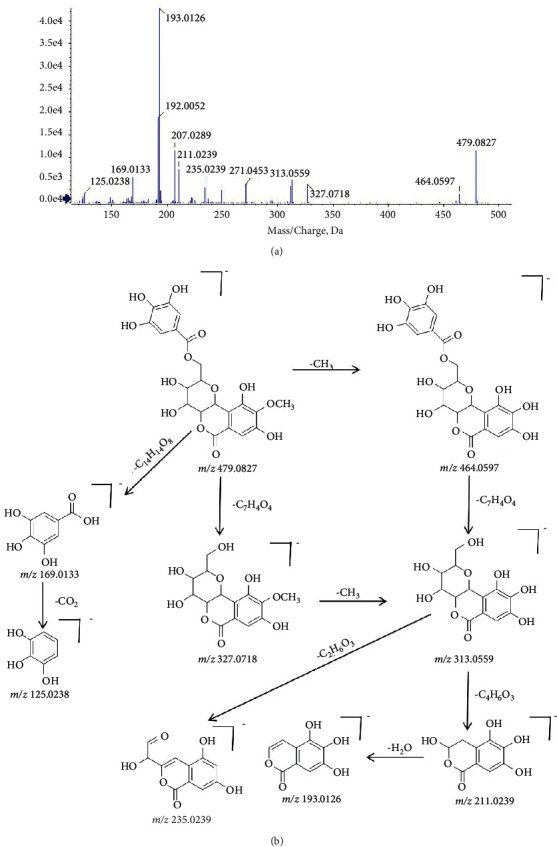
The mass spectrum and the cleavage rule of 11-O-galloylbergenin.

Take 11-O-galloylbergenin (compound 25) as an example to explain the process of identification. Compound 25 showed a favorable response in both positive ion and negative ion mode. The quasimolecular ion [M − H]^−^ at *m/z* 479.1 corresponded to the formula C_21_H_20_O_13_. The fragment ion *m/z* 464.1 was formed by the loss of methyl. The ester bond of the parent ion was cleaved to form a gallic acid fragment at *m/z* 169.0 [M − H-C_14_H_14_O_8_]^−^and a bergenin fragment at *m/z* 327.1 [M − H-C_7_H_4_O_4_]^−^. The fragment ion [M − H-C_15_H_14_O_10_]^−^ was formed by the loss of CO_2_ from the gallic acid fragment. Demethylbergenin fragment *m/z* 313.1 [C_13_H_13_O_9_]^−^ was produced due to the loss of methyl by the bergenin fragment. The bergenin fragment was cleaved to produce fragments *m/z* 235.0, *m/z* 211.0, and 193.0. Combined with literature [29], compound 25 was speculated to be 11-O-galloylbergenin. The mass spectrum cracking rule of compound 25 is shown in Figure 4.

#### 3.1.3. Identification of Amino and Its Derivatives in KHJ

Seven nitrogenous compounds from Cicadae Periostracum were identified from KHJ and speculated to be amino acids and acetyldopamine dimers. Amino acid was the main component, and acetyldopamine dimer was the main anti-inflammatory and antioxidant component of Cicadae Periostracum [24].

**Figure 5:**
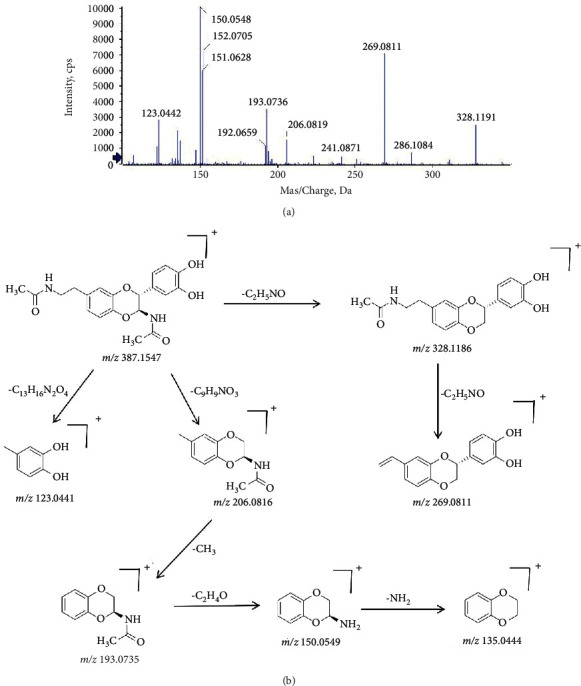
The mass spectrum and the cleavage rule of (2R,3S)-2-(3′,4′-dihydroxyphenyl)-3-acetylamino-7-(N-acetyl-2″-aminoethyl)-1,4-benzodioxane.

Take (2R,3S)-2-(3′,4′-dihydroxyphenyl)-3-acetylamino-7-(N-acetyl-2″-aminoethyl)-1,4-benzodioxane (compound 26) and (2R,3S)-2-(3′,4′-dihydroxyphenyl)-3-acetylamino-6-(N-acetyl-2″-amino-1″-hydroxyethyl)-1,4-benzodioxane (compound 27) as examples for the interpretation of structure analysis. Compounds 26 and 27 were isomers with the molecular formula of C_20_H_22_N_2_O_6_ based on the quasimolecular ion [M + H]^+^ at *m/z* 387.2. The MS^2^ fragmental ions at *m/z* 328.1, *m/z* 269.1, and *m/z* 206.1 were obtained due to the loss of C_2_H_5_NO, C_4_H_10_N_2_O_2_, and C_9_H_9_NO_3_. According to the retention time in literature [20], compounds 26 and 27 were identified. The mass spectrum and possible cleavage pathways of compound 26 are shown in Figure 5.

#### 3.1.4. Identification of Flavonoids in KHJ

**Figure 6:**
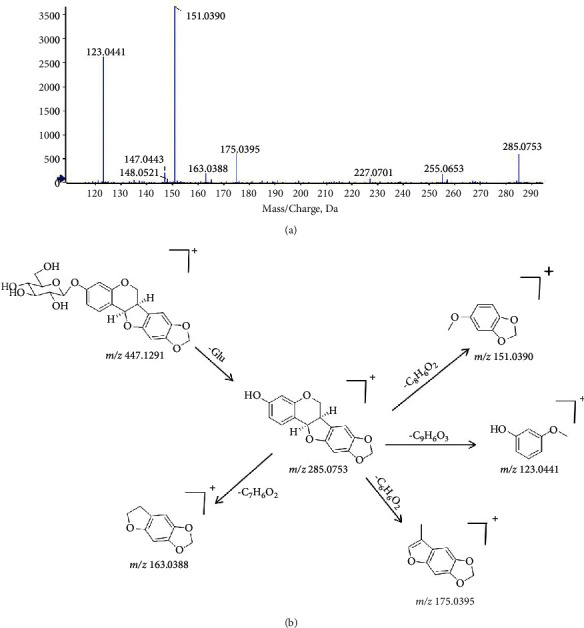
The mass spectrum and the cleavage rule of trifolirhizin.

Eight flavonoids from Sophorae Tonkinensis Radix were identified in KHJ, including dihydroisoflavones and pterocarpin. Compared with dihydroisoflavones, pterocarpin compounds were more compact in structure and less prone to RDA rearrangement. It is reported that flavonoids from Sophorae Tonkinensis Radix have an effect on anti-inflammatory, antibacterial, and other biological activities. Trifolirhizin (compound 39) was used as a sample to expound the process of analysis. The formula C_22_H_22_O_10_ was confirmed due to the quasimolecular ions [M + Na]^+^ at *m/z* 469.1 and [M + HCOOH–H]^−^ at *m/z* 491.1. The fragment ion *m/z* 285.1 was formed due to the loss of glucose reside. The fragment ions *m/z* 151.0 [M + H-Glc-C_8_H_6_O_2_]^+^ and 123.0 [M + H-Glc–C_8_H_6_O_2_–CO]^+^ were produced by the cleavage of the C ring and loss of CO. Thus, compound 39 was identified to be trifolirhizin. The mass spectrum and possible cleavage pathways of compound 39 are shown in Figure 6.

#### 3.1.5. Identification of Saponin in KHJ

**Figure 7:**
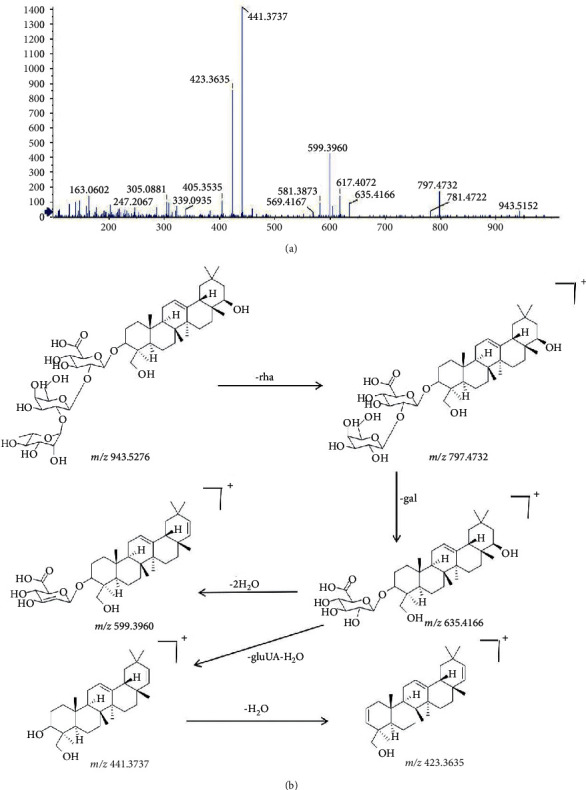
The mass spectrum and the cleavage rule of soyasaponin I.

Nine saponins were identified from KHJ. Among them, subprosides V, subproside II methyl ester, soyasaponin I, and kudzusaponin A3 were isolated from Sophorae Tonkinensis Radix, and ardisicrenoside B, ardisicrenoside H, ardisicrenoside *G*, ardisicrenoside N, and ardisiacrispin A were isolated from Ardisiae Radix. Take soyasaponin I (compound 52) as an example to explain the process of compound analysis. The quasimolecular ion [M + H]^+^ at *m/z* 943.5 corresponded to the formula C_48_H_78_O_18_. The fragment at *m/z* 797.5, 635.4, and 599.4 was produced by the successive loss of a rhamnose residue, galactose residue, and two H_2_O from the parent ion. The fragment at *m/z* 441.3 and 423.4 was formed by the successive loss of H_2_O from aglycone. Thus, compound 52 was speculated as soyasaponin I. The mass spectrum cracking rule of compound 52 is shown in Figure 7.

### 3.2. Cytotoxicity of KHJ against RAW264.7 Cells

The cytotoxicity of the regularity of recipe composition samples and the whole prescription sample was determined by MTT assay [30]. The results showed that KHJ had no cytotoxicity on RAW 264.7 cells at the doses of 1–100 *μ*g/mL. The cell survival rate of KHJ-2, KHJ-4, and KHJ-9 was significantly lower than that of the blank group (*P* < 0.05) at the dose of 200 *μ*g/mL, indicating that KHJ-2, KHJ-4, and KHJ-9 at the concentration of 200 *μ*g/mL could inhibit the proliferation of RAW264.7 cells.

### 3.3. Inhibition Effect of KHJ on NO Production in LPS-Induced RAW 264.7 Cells

**Figure 8:**
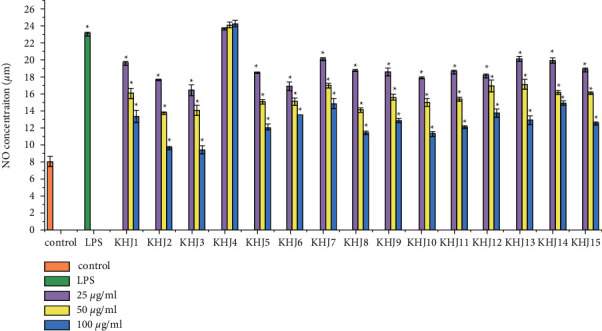
Inhibition effect of KHJ on NO production in LPS-induced RAW 264.7 cells.

As shown in Figure 8, LPS-induced RAW264.7 cells can significantly promote the production of NO, and the content of NO in KHJ-4 had no significant difference compared with that in the model group (*P* > 0.05). Other groups could inhibit the release of NO in RAW264.7 cells to different degrees (*P* > 0.05).

The IC value was calculated by IBM SPSS 23.0 and converted into the concentration of the samples. As shown in Table 3, KHJ-2, KHJ-9, KHJ-5, KHJ-11, and KHJ-1 groups had a stronger anti-inflammatory effect in vitro. These results indicated that the anti-inflammatory active ingredients of KHJ mainly came from Sophorae Tonkinensis Radix and Ardisiae Radix.

### 3.4. Gray Correlation Analysis

**Figure 9:**
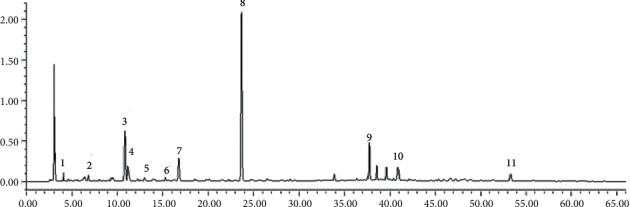
Eleven main peaks in KHJ.

Gray relational analysis (GRA) was a quantitative description and comparison method for the development and change of a system. Its basic idea was to judge whether the relationship was close by determining the geometric shape similarity between the reference data column and several comparative data columns, which reflects the correlation degree between the curves. In this study, the spectrum-effect relationship of KHJ was studied through the GRA method. A total of 11 main peaks were selected according to the HPLC-DAD analysis for the regularity of recipe composition samples (Figure 9). The anti-inflammatory activity score of each sample was taken as a reference column, and the correlation degree was calculated after the original data was treated with the dimensionless standard. The results of peak area and spectrum-effect correlation analysis are shown in Table 4.

As shown in Table 4, the correlation degree of all peaks was greater than 0.5. This indicated that ingredients in KHJ were acting in synergy. The ranking of correlation degree was peak 1 > 6>9 > 8>7 > 10>4 > 5>11 > 3>2. Among them, peaks 2, 4, 5, 6, 8, 9, and 11 were identified as anagyrine, matrine, sophocarpine, norbergenin, bengenin, 11-O-galloylbergenin, and trifolirhizin through HPLC/Q-Tof-MS/MS analysis. These ingredients could be candidates for the Q-Markers of KHJ on anti-inflammation.

### 3.5. Network Pharmacological Analysis

**Figure 10:**
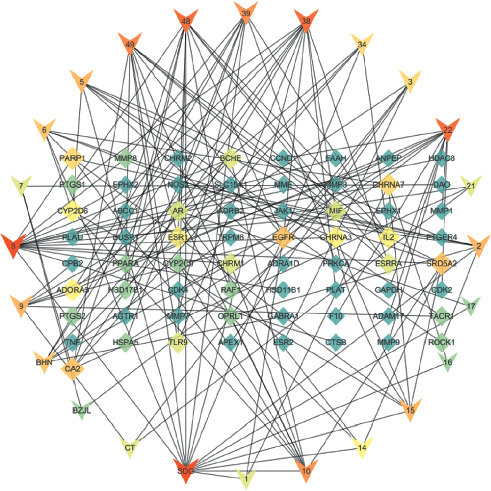
Ingredient-target network.

**Figure 11:**
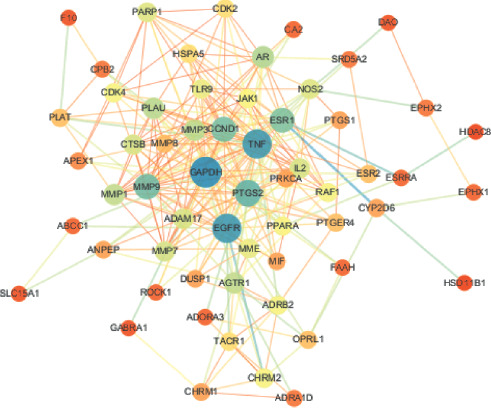
PPI network.

**Figure 12:**
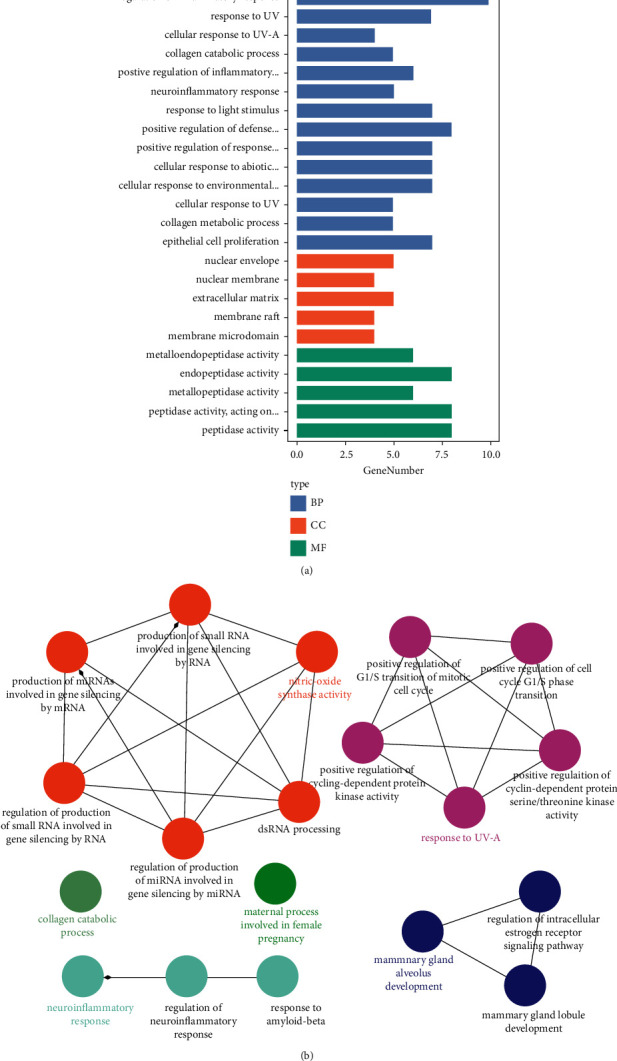
GO enrichment analysis.

As shown in Figure 10, the active ingredient of the drug-potential target was visualized by Cytoscape 3.7.0. The key active ingredients, which are higher than the average value and the core target of KHJ, were imported into the STRING database and processed by Cytoscape 3.7.0 to obtain the PPI network (Figure 11). The network consists of 65 nodes and 293 edges. The targets whose values were greater than the average degree value were as follows: GAPDH, EGFR, TNF, PTGS2, MMP 9, CCND1, ESR1, AR, PLAU, MMP3, AGTR1, MMP1, ADAM17, IL2, CTSB, MMP7, NOS2, PARP1, MME, and TLR9. Metascape database (https:metascape.org) was used for GO enrichment analysis and KEGG analysis of 20 key targets. The 20 key targets were mainly involved in biological functions such as ultraviolet response, inflammation regulation, collagen catabolism process, light stimulation response, nerve inflammation response, and external stimulation response by influencing the activities of metalloendopeptidase, serine proteolytic enzyme, and serine-type endopeptidase. The results are shown in Figure 12(a). The 20 key targets were analyzed for GO enrichment by ClueGO. As shown in Figure 12(b), 20 key targets of KHJ were mainly involved in the nitric oxide synthase activity signal transduction pathway, ultraviolet radiation response signal pathway, nerve inflammation response, and collagen catabolism process.

**Figure 13:**
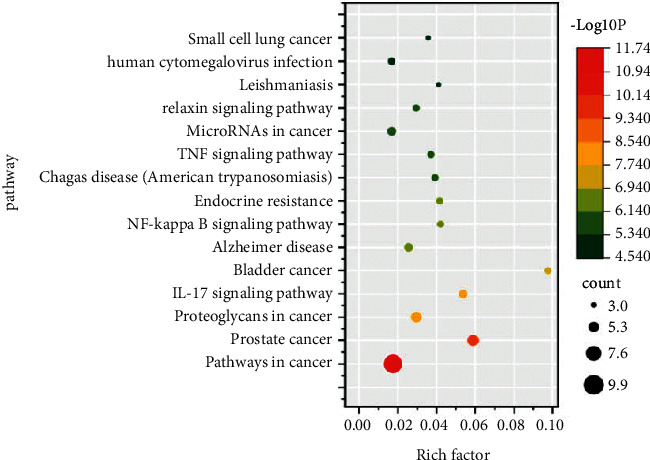
KEGG analysis.

The Metascape database was used for KEGG analysis, involving 61 entries, in which the cancer pathway, prostate cancer pathway, and interleukin-17 signal pathway were enriched with more genes and smaller *P* value. These results are shown in Figure 13.

**Figure 14:**
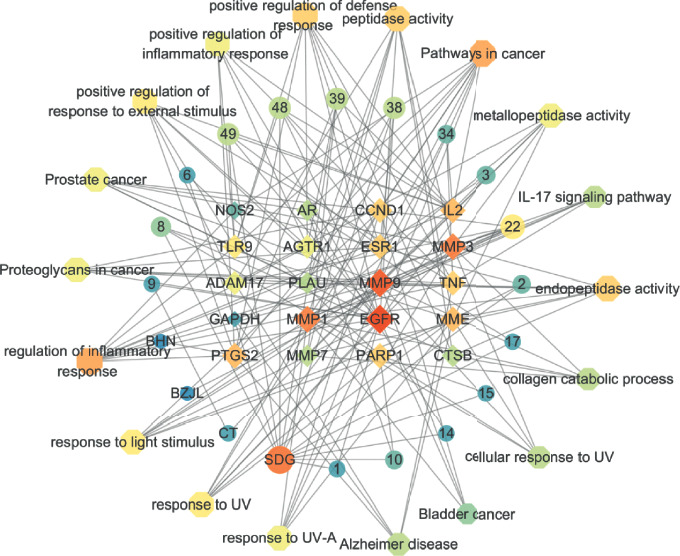
Compound-target-pathway network.

The data of compounds, targets, and pathways were imported into Cytoscape 3.7.0 to obtain a network, which contains 58 nodes and 175 edges, and the nodes increased with the degree value. The results suggested that KHJ may exert an anti-inflammatory effect through multicomponent and multitarget. Bergenin, matrine, sophocarpine, calycosin, and trifolirhizin were the main anti-inflammatory active ingredient in KHJ. The main targets of KHJ were EGFR, MMP9, MMP3, MMP1, and PTGS2. The results are shown in Figure 14.

## 4. Conclusion

Miao medicine was an important part of TCM. However, similar to TCM, the lack of quality standards also seriously restricted the standardization and modernization of Miao medicine. The proposal of Q-Marker pointed out the direction for the quality research of TCM. However, how to discover and identify the Q-Marker was still a great challenge for TCM and Miao medicine.

In this paper, a gray correlation analysis strategy combined with network pharmacology analysis was proposed to investigate the Q-Markers of KHJ. The results show that bergenin, sophocarpidine, sophocarpine, and trifolirhizin could be regarded as the Q-Markers of KHJ on anti-inflammation. The process of discovering the Q-Markers would provide a promising method of quality control on KHJ. Nevertheless, the specific contribution of each Q-Marker in the formulation had not been clarified, which needs to be further investigated.

## Figures and Tables

**Table 1:** Ingredients of each group.

No.	Sophorae Tonkinensis Radix	Ardisiae Radix	Cicadae Periostracum	Menthol
KHJ1	+	−	−	−
KHJ2	−	+	−	−
KHJ3	−	−	+	−
KHJ4	−	−	−	+
KHJ5	+	+	−	−
KHJ6	+	−	+	−
KHJ7	+	−	−	+
KHJ8	−	+	+	−
KHJ9	−	+	−	+
KHJ10	−	−	+	+
KHJ11	+	+	+	−
KHJ12	+	+	−	+
KHJ13	+	−	+	+
KHJ14	−	+	+	+
KHJ15	+	+	+	+

**Table 2:** Identification results of chemical ingredients in KHJ.

No.	Molecular formula	*t*_*R*_ (min)	Calcd (*m/z*)	Exptl (*m/z*)	*δ /ppm*	Ion mode	MS/MS	Identification	Sources
1	C_12_H_16_N_2_O	4.67	205.1335	205.1328	3.41	[M + H]^+^	205.1314; 146.0603; 108.0809; 162.0920	N-Methylcytisine	STR
2	C_15_H_22_N_2_O_2_	5.41	263.1754	263.1753	0.38	[M + H]^+^	263.1753; 150.1276; 195.1491; 245.1649	(5*α*/12*α*/12*β*)-Hydroxysophocarpine	STR
3	C_9_H_11_NO_3_	5.49	182.0812	182.0812	0	[M + H]^+^	119.0492; 107.0493; 123.0442; 136.0761	Tyrosine	CPS
4	C_15_H_24_N_2_O_2_	5.99	265.1911	265.1914	−1.13	[M + H]^+^	265.1914; 219.1862; 148.1123; 247.1815	(9*α*/5*α*)-Hydroxymatrine	STR
5	C_15_H_24_N_2_O_2_	6.61	265.1899	265.1899	0	[M + H]^+^	265.1899; 150.1271; 247.1820; 112.0754	14*α*-Hydroxymatrine	STR
6	C_15_H_20_N_2_O	7.03	245.1648	245.1641	2.86	[M + H]^+^	245.1649; 122.0601; 148.0760	Anagyrine	STR
7	C_7_H_6_O_5_	7.7	169.0143	169.0137	3.5	[M − H]^−^	125.0239; 107.0136	2,4,6-Trihydroxybenzoic	AR
8	C_15_H_24_N_2_O	9.26	249.1961	249.1951	4.01	[M + H]^+^	249.1957; 148.1109; 176.1065; 112.0754	Matrine	STR
9	C_9_H_11_NO_2_	9.66	166.0863	166.0862	0.6	[M + H]^+^	103.0541; 120.0809	L-Phenylalanine	CPS
10	C_15_H_22_N_2_O	10.56	247.1805	247.1793	4.85	[M + H]^+^	247.1793; 136.1116; 179.1537; 150.1277	Sophocarpine	STR
11	C_17_H_26_N_2_O_3_	11.12	307.2016	307.2021	−1.62	[M + H]^+^	247.1816; 307.2034; 148.1124	(14*β*/14*α*)-acetyl matrine	STR
12	C_17_H_26_N_2_O_3_	11.8	307.2016	307.2027	−3.58	[M + H]^+^	307.2027; 247.1812; 148.1123; 176.1071	(14*β*/14*α*)-acetyl matrine	STR
13	C_13_H_14_O_9_	12.26	313.0565	313.0562	1	[M − H]^−^	193.0133; 207.0293; 165.0187; 235.0246	Norbergenin	AR
		12.34	315.0711	315.0709	0.63	[M + H]^+^	167.0341; 191.0346; 177.0183; 207.0296		
14	C_15_H_22_N_2_O_2_	12.42	263.1764	263.1764	0	[M + H]^+^	245.1655; 263.1764; 150.1278; 136.1121	Oxysophocarpine	STR
15	C_15_H_20_N_2_O	14.4	245.1648	245.1648	0	[M + H]^+^	110.0602; 136.1123; 180.9101; 245.1663	Sophoramine	STR
16	C_12_H_14_N_2_O_2_	15.45	219.1128	219.1129	−0.46	[M + H]^+^	148.0756; 160.0757; 219.1133; 191.1183	N-Formylcytisine	STR
17	C_15_H_22_N_2_O_3_	15.8	279.1703	279.1708	−1.79	[M + H]^+^	261.1603; 164.1073; 149.0234; 243.1499	5*α*-Hydroxyoxysophocarpine	STR
18	C_10_H_13_NO_3_	17.58	196.0968	196.0969	−0.51	[M + H]^+^	119.0487; 137.0594; 109.0647; 107.0491	N-Acetyldopamine	CPS
19	C_15_H_22_N_2_O_3_	18.72	279.1703	279.1714	−3.94	[M + H]^+^	279.1714; 149.0236; 243.1504; 261.1606	12*β*-Hydroxyoxysophocarpine	STR
20	C_13_H_16_N_2_O_2_	18.88	233.1285	233.1289	−1.72	[M + H]^+^	148.0759; 191.1180; 233.1289; 160.0758	N-Acetylcytisine	STR
21	C_7_H_6_O_3_	19.21	137.0244	137.0238	4.38	[M − H]^−^	108.0211; 136.0161; 137.0240; 109.0284	3,4-Dihydroxybenzaldehyde	CPS
22	C_14_H_16_O_9_	21.31	327.0722	327.0714	2.44	[M − H]^−^	192.0049; 193.0131; 234.0159; 164.0109	Bergenin	AR
		21.37	329.0867	329.0864	0.91	[M + H]^+^	179.0346; 181.0498; 193.0490; 197.0447		
23	C_21_H_20_O_9_	28.5	415.1035	415.1046	−2.65	[M − H]^−^	267.0655; 295.0607	Bayin	STR
24	C_27_H_30_O_13_	28.74	563.1759	563.1761	−0.36	[M + H]^+^	417.1185; 399.1076; 297.0761; 381.0976	Sophoraflavone A	STR
25	C_21_H_20_O_13_	33.58	481.0977	481.0969	1.66	[M + H]^+^	153.0178; 181.0499; 209.0449; 275.0560	11-O-Galloylbergenin	AR
		33.6	479.0831	479.0805	0.83	[M − H]^−^	193.0126; 479.0827; 169.0133		AR
26	C_20_H_22_N_2_O_6_	35.04	387.1551	387.1545	1.55	[M + H]^+^	150.0549; 193.0735; 269.0811; 328.1185	(2R,3S)-2-(3′,4′-Dihydroxyphenyl)-3-acetylamino-7-(N-acetyl-2″-aminoethyl)-1,4-benzodioxane	CPS
27	C_20_H_22_N_2_O_6_	36.77	387.1551	387.1544	1.81	[M + H]^+^	150.0549; 193.0735; 269.0811; 328.1185	(2R,3S)-2-(3′,4′-Dihydroxyphenyl)-3-acetylamino-6-(N-acetyl-2″-amino-1″-hydroxyethyl)-1,4-benzodioxane	CPS
28	C_21_H_20_O_10_	37.21	433.1129	433.1136	−1.62	[M + H]^+^	271.0604; 164.9305; 243.0655	Vitexin	STR
		37.27	431.0984	431.0996	−2.78	[M − H]^−^	269.0460; 186.9376; 119.9454		
29	C_28_H_36_O_13_	39.35	579.2083	579.2099	−2.76	[M − H]^−^	417.1562; 181.0501	Syringaresinol-4-O-*β*-*D*-glucopyranoside	STR
30	C_20_H_20_N_2_O_6_	40.35	407.1219	407.1218	0.25	[M + Na]^+^	150.0551; 204.0660; 284.0919; 122.0599	(2R,3S)-2-(3′,4′-Dihydroxyphenyl)-3-acetylamino-7-(N-acetyl-2″-aminoethyl-ene)-1,4-benzodioxane	CPS
31	C_20_H_20_N_2_O_6_	43.64	385.1394	385.14	−1.56	[M + H]^+^	150.0549; 249.0551; 284.0926; 239.0704	(2R,3S)-2-(3′,4′-Dihydroxyphenyl)-3-acetylamino-7-(N-acetyl-2″-aminoethyl)-1,4-benzodioxane	CPS
32	C_22_H_22_O_12_	44.42	479.1184	479.1187	−0.63	[M + H]^+^	151.0391; 209.0452; 181.0500; 247.0604	11-O-Vanilloyl-bergenin	AR
		44.54	477.1039	477.1018	4.4	[M − H]^−^	192.0061; 207.0293; 234.0166; 164.0108		
33	C_23_H_24_O_13_	44.81	509.129	509.1303	−2.55	[M + H]^+^	181.0497; 209.0454; 275.0547; 153.0544	11-O-Syringyl-bergenin	AR
34	C_22_H_22_O_9_	47.5	431.1337	431.1340	−0.7	[M + H]^+^	269.0813; 254.0578	Ononin	STR
35	C_23_H_24_O_13_	49.03	509.129	509.129	0	[M + H]^+^	181.0498; 209.0449; 275.0561	*11-O-(3′,4′-*Dimethyl-galloyl)bergenin	AR
		49.18	507.1144	507.112	4.73	[M − H]^−^	192.0059; 207.0297; 234.0166		
36	C_54_H_88_O_24_	49.91	1143.5563	1143.5576	−1.17	[M + Na]^+^	439.3604; 457.3699; 421.3476; 615.3938	Subprosides V	STR
37	C_52_H_84_O_23_	50.58	1099.5294	1099.5283	1	[M + Na]^+^	—	Ardisicrenoside H	AR
		50.65	1075.5331	1075.5309	2.05	[M − H]^−^	1076.5404; 1075.5372; 943.4975		
38	C_16_H_12_O_5_	51.21	285.0758	285.0762	−1.34	[M + H]^+^	270.0535; 253.0496; 137.0234; 225.0547	Calycosin	STR
		51.32	283.0612	283.0615	−1.06	[M − H]^−^	268.0374; 211.0396; 239.0342; 135.0083		
39	C_22_H_22_O_10_	53.82	469.1107	469.11	2.28	[M + Na]^+^	151.0390; 123.0441; 285.0753; 175.0395	Trifolirhizin	STR
		53.92	491.1195	491.1198	−0.61	[M + HCOOH–H]^−^	104.9532; 146.9651; 283.0603; 325.1873		
40	C_23_H_24_O_12_	53.89	491.1195	491.1182	2.65	[M − H]^−^	283.0606; 255.0658; 192.0061	11-O-3,5-Dimethoxybenzoyl bergenin	AR
41	C_48_H_78_O_20_	56.77	975.5159	975.5179	−2.05	[M + H]^+^	473.3640; 455.3536; 437.3424; 631.3871	Kudzusaponin A3	STR
		56.82	973.5014	973.5052	−3.9	[M − H]^−^	973.5052; 701.8178; 401.1368		STR
42	C_52_H_86_O_22_	57.69	1085.5494	1085.5489	0.46	[M + Na]^+^	—	Ardisicrenoside B	AR
		57.74	1107.5593	1107.5641	−4.33	[M + HCOOH–H]^−^	1062.5634; 1061.5581; 929.5087		
43	C_48_H_78_O_19_	59.02	959.521	959.5229	−1.98	[M + H]^+^	439.3615; 959.5621	Subproside II methyl ester	STR
44	C_24_H_24_O_11_	63.21	511.1216	511.1215	0.2	[M + Na]^+^	151.0393; 123.0444; 285.0762; 175.0398	Trifolirhizin 6′-monoacetate	STR
45	C_53_H_86_O_23_	63.92	1113.5464	1113.5452	1.08	[M + Na]^+^	—	Ardisicrenoside G	AR
		64.75	1089.5487	1089.5336	−4.50	[M − H]^−^	927.5146; 765.4486		
46	C_52_H_84_O_22_	64.74	1105.5436	1105.5469	−2.98	[M + HCOOH–H]^−^	1060.5409; 1059.5383; 927.4984; 765.4458	Ardisiacrispin A	AR
		64.74	1083.5312	1083.531	0.18	[M + Na]^+^	1083.5311; 1084.5362; 455.3527; 295.1033		
47	C_53_H_86_O_22_	65.18	1097.5487	1097.5481	0.55	[M + Na]^+^	—	Ardisicrenoside N	AR
		65.26	1119.5593	1119.5625	−2.86	[M + HCOOH–H]^−^	1074.5578; 1073.5558; 927.4964		
48	C_16_H_12_O_4_	65.8	269.0808	269.0812	−1.49	[M + H]^+^	269.0817; 253.0501; 226.0632; 197.0602	Formononetin	STR
		65.85	267.0663	267.0663	0	[M − H]^−^	252.0426; 223.0397; 195.0444; 132.0213		
49	C_17_H_14_O_5_	65.94	299.0914	299.0928	−4.68	[M + H]^+^	284.0689; 299.0932; 256.0739; 241.0499	8-O-Methylretusin	STR
50	C_17_H_14_O_5_	66.83	299.0914	299.0923	−3.01	[M + H]^+^	284.0689; 299.0932; 256.0739	Pterocarpin	STR
51	C_26_H_26_O_11_	68.9	537.1373	537.1381	−1.49	[M + Na]^+^	165.0543; 231.0654; 285.0741; 375.0871	Sophoratonkin	STR
52	C_48_H_78_O_18_	71.41	943.5261	943.5276	−1.59	[M + H]^+^	441.3737; 599.3960; 797.4732	Soyasaponin I	STR
		71.47	941.5115	941.5149	−3.61	[M − H]^−^	941.5149; 514.3251; 311.1685		

Notes: STR: Sophorae Tonkinensis Radix; AR: Ardisiae Radix; CPS: Cicadae Periostracum; MEL: menthol.

**Table 3:** Anti-inflammatory effect of KHJ.

No.	IC50 value (crude drug *μ*g/mL)	Score of anti-inflammatory effect
KHJ1	742.02	11
KHJ2	306.67	15
KHJ3	1674.16	3
KHJ4	—	—
KHJ5	683.86	13
KHJ6	875.4	8
KHJ7	990.67	6
KHJ8	821.13	9
KHJ9	450.51	14
KHJ10	2180.11	2
KHJ11	719.08	12
KHJ12	941.07	7
KHJ13	1303.93	5
KHJ14	1456.57	4
KHJ15	788.03	10

**Table 4:** Peak area and correlation analysis results of each chromatographic peak.

No.	Peak area										
	1	2	3	4	5	6	7	8	9	10	11
KHJ1	337675	3250741		7883555	1361583		6562137				3438815
KHJ2	131096		3549827			1194809	2984297	29924270	8249006	2666166	
KHJ3										1166193	
KHJ5	269484	338361	2194995	3945104	655154	672761	4204828	21735193	3965420	1601346	1400291
KHJ6	367008	131227		3761086	666981		3197515			2293113	1772313
KHJ7	287472	2647413		6463130	1140807		5399129				2891330
KHJ8	225653		15812017			425171	493058	24402042	2135026	3281601	
KHJ9	124643		3316416			1118268	2705511	29051519	7515400	2453783	
KHJ10										1591425	
KHJ11	233145	521181	5757911	2261501	409749	424601	3120952	20053990	3172558	2355283	1200859
KHJ12	254038	104866	1791561	3318848	586303	448102	3736736	17817825	3334396	1317748	1176199
KHJ13	449347	121253		4832987	952655		4008906			2999136	2504868
KHJ14	175440		13509058			402603	565591	22001498	2226591	3614359	
KHJ15	244968	566730	5256412	2078015	406265	369635	2921097	19315474	2644608	2116705	1088261
Correlation degree	0.721	0.55	0.577	0.648	0.645	0.713	0.698	0.707	0.712	0.682	0.638

## Data Availability

All data used to support the findings of this study are included within this paper.
